# Mechanics and physics of a glass/particles photonic sponge

**DOI:** 10.1038/s41598-020-75504-9

**Published:** 2020-11-11

**Authors:** M. Dubernet, E. Bruyer, Y. Gueguen, P. Houizot, J. C. Hameline, X. Rocquefelte, T. Rouxel

**Affiliations:** 1 Institut de Physique de Rennes, IPR, UMR UR1-CNRS 6251, Université de Rennes 1, 35042 Rennes Cedex, France; 2 Institut Des Sciences, Chimiques de Rennes-UMR 6226, CNRS, University of Rennes, 35000 Rennes, France; 3 Institut Universitaire de France, Paris, France

**Keywords:** Materials science, Condensed-matter physics, Materials for optics, Structural materials, Theory and computation, Mechanical engineering

## Abstract

A glass containing mechanoluminescent crystalline particles behaves as a photonic sponge: that is to say it fills up with trapped electrons when exposed to UV light, and it emits light when submitted to a mechanical loading, similar to a sponge soaked with water that is wringed under mechanical action! A major finding of the present study is that the elasto-mechanoluminescence effect showing up on unloading is governed by the deviatoric part of the applied stress (no effect under hydrostatic pressure). Furthermore, the structural source for this phenomenon was elucidated by a detailed density functional theory analysis of the e^−^ energetics at the possible oxygen vacancy sites within the crystalline phase. Both the e^−^ trapping and detrapping processes under load could be explained. An analogy with hydraulic circuits and the rheology of viscoelastic media was successfully introduced to pave the way to a constitutive law for the mechano-optical coupling phenomenon.

## Introduction

Several compounds are known to emit light under mechanical loading, especially in the elastic regime^1–5^. This is the elasto-mechanoluminescence (EML) phenomenon. This property opens a new realm of possibilities for innovative applications in various domains, such as the development of free-standing lightning devices, surface damage detection in security devices, or for attracting someone's attention in a dark environment to mention a few of them we have identified. Nevertheless, the mechanics and the structural source of EML still remain poorly understood^4^. The EML intensity, I_EML_, depends on the loading rate and loading configuration (compression, shear, hydrostatic etc.). A major finding of previous investigations^6,7^ is that I_EML_ scales with the mechanical power, but the actual influence of the loading history and the occurrence of delayed effects are not elucidated yet. In most cases, the emitted light covers a narrow wavelength interval that can be attributed to some specific electronic transitions, such as the f → d one in compounds containing rare-earth elements, and the delayed emission would stem from the de-excitation of electrons (e^−^) being trapped at some structural defect sites (oxygen vacancies for example)^8,9^, thanks to thermal activation, as in the case of phosphorescence (PL), and to the combination of thermal activation and mechanical loading as for EML. At the structural scale, it was proposed^10^ that the stress-induced detrapping phenomenon would result from the occurrence of a local electric field under stress, especially in non-centrosymetric crystals. In this study a transparent glass-matrix particulate composite exhibiting EML was submitted to series of mechanical testing experiments, with the aim to clarify the dependence of I_EML_ on the loading configuration and history. In parallel, the active defects of the crystalline particles were identified and density functional theory (DFT) calculations were performed to determine the energy levels and electronic band structures associated with the defects, and further to investigate the sensitivity of these sites to mechanical loading.

## Materials and method

The studied material is a glass-matrix particulate composite consisting of a (Li,Na)-phosphate glass (Li_2_O(25%)–Na_2_O(25%)–P_2_O_5_(50%)), referred to as NaPOLi, in which 3 vol.% SrAl_2_O_4_:Eu,Dy particles^11^ about 10 µm in size were incorporated. The composite was obtained by means of a five step process: (1) mixing of NaH_2_PO_4_, LiH_2_PO_4_, and SrAl_2_O_4_:Eu,Dy particles in a platinum crucible (raw materials from Sigma-Aldrich (DE), > 99% purity); (2) de-hydration at 573 K for 2 h; (3) melting at 1073 K for 3 min; (4) pouring and shaping in a stainless steel mould (316 L) preheated at 523 K (~ T_g_ of the glass); (5) annealing at T_g_ for two hours and slow cooling (< 2 K min^−1^) to room temperature.

Mechanical testing experiments were carried out on 4(width) × 4(depth) × 4.5(height) mm^3^ specimens, in compression by means of an home-made testing machine (see ref^12^ for details), consisting of a piezoelectric actuator (N-216 Nexline, Phys. Instr. Co. (DE)), a stiff load cell (MS02, Scaime Co. (F)), equipped with a high sensitivity camera (Zyla sCMOS 5.5, Andor Tech. Ltd (Belfast, IR)) in order to record the EML intensity. The imposed displacement rate lies between 0.01 µm s^−1^ and 400 µm s^−1^. The displacement is measured by means of a laser interferometer (LK-G5000, Keyence Co. (JP)) with a ± 5 nm accuracy. In addition, some high-pressure experiments were carried out at pressure up to 100 MPa, in a helium gas chamber with a sapphire glass window allowing for the Zyla camera to be used. In this latter case, pressure is applied within a few seconds and maintained for about 2 min. Specimens were exposed to UV light (365 nm) for 30 s prior to mechanical testing (this is how the "sponge" is filled prior to testing) and the loading cycle started 10 min later, that is when the phosphorescence background emission became negligible in comparison with the EML intensity.

The DFT calculations were performed using the VASP code (Vienna ab-initio Simulation Package; www.vasp.at)^13,14^, considering the ambient pressure unit cell of the P2_1_ space group SrAl_2_O_4_ crystal, with *a* = 8.447 Å, *b* = 8.816 Å, *c* = 5.163 Å and *β* = 93.42°^15^. The VASP code allows the determination of the ground state properties for a given compound based on DFT calculation and is based on a plane wave based Projector Augmented Wave (PAW) approach. The semi-local Perdew–Burke–Ernzerhof (PBE) functional^16^ on a (2a,2b,3c) supercell containing 336 atoms was used to identify the defect energy levels responsible for the detrapping process under mechanical loading and unloading. The wavefunctions were expanded in plane waves up to a kinetic energy cut-off of 400 eV, and the k-point sampling was chosen sufficiently fine to ensure the numerical convergence of all the calculated properties. In particular, the accurate calculation of the total energy for all defect models (336 atoms supercell) was carried out using a 6 × 6 × 6 k-point mesh. The geometry was optimized until the forces on all the atoms were less than 3.10^–4^ eV Å^−1^. It should be noticed that the use of the PBE functional leads to a significant underestimation of the band gap: a value of 4.2 eV is obtained while a band gap of 6.5 eV was reported from experiments^17^. However, keeping in mind that the purpose of the present calculations is to estimate the energy depth of the oxygen vacancies with respect to the conduction band, all conclusions drawn using the PBE approximation are assumed to be valid.

## Results and discussion

### The photonic "sponge" and the way it is wringed out

**Figure 1:**
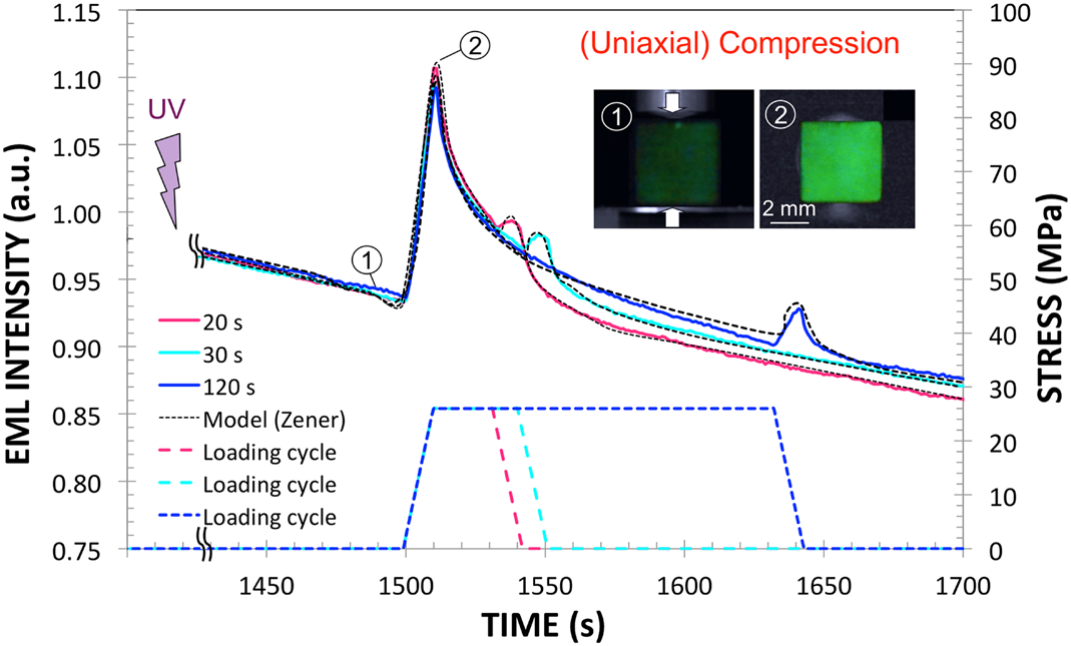
Uniaxial compression test, with a preliminary exposure to UV light (365 nm) for 30 s. A stress of 26 MPa was applied for 20, 30 and 120 s, at time t = 1500 s post UV exposure, after the phosphorescence background light became weak enough to allow for the study of the mechanoluminescence on a (4 × 4 × 4.47 mm^3^ parallelepiped sample). The black dotted curves show the data fit obtained with the Zener rheological model (see text for details). A video showing the elasto-mechanoluminescence phenomenon observed in compression is provided as a supplementary material (video 1).

**Figure 2:**
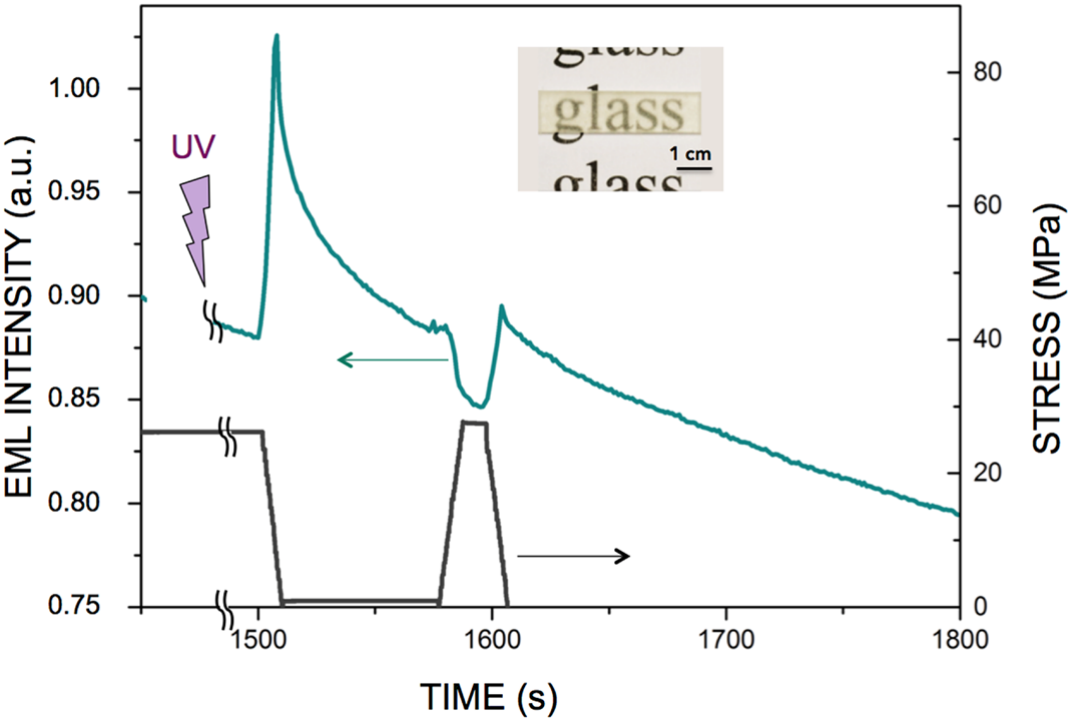
Uniaxial compression test, with the preliminary UV irradiation being performed under load. A 4 × 4 × 4.47 mm^3^ parallelepiped sample was UV-irradiated for 30 s under a 26 MPa compression load. The sample was loaded for 1500 s, unloaded at 1500 s and further reloaded at 1575 s for 10 s. The inset shows the bar from which were cut the specimens for mechanical testing.

**Figure 3:**
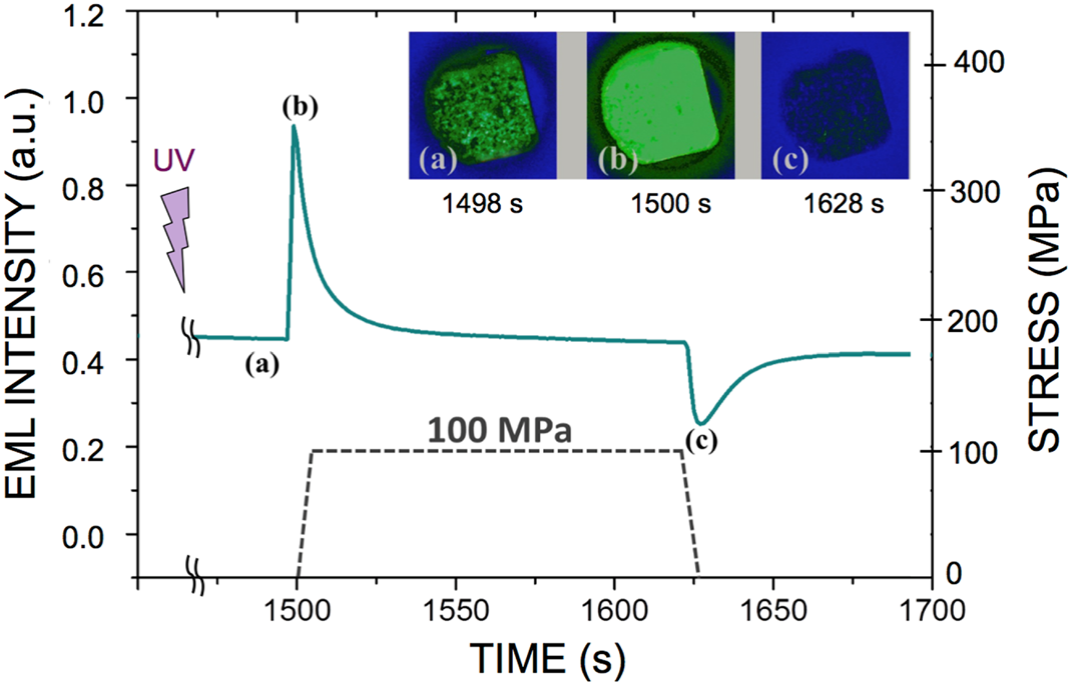
Hydrostatic pressure experiment. A parallelepiped sample (2.4 × 3.1 × 4 mm^3^) was placed in a chamber filled with helium gas and observed through a sapphire window. The specimen was irradiated with a UV-light source (365 nm) for 300 s prior to testing. Pressure was applied 1500 s later. Note that there is no increase (a decrease is observed instead) of the mechanoluminescence intensity upon unloading at t = 1625 s. The inset pictures show a partial view of the sample (real color) at positions **a**, **b** and **c** on the intensity curve respectively.

An interesting finding of this study is that EML shows up not only on loading but on unloading as well (Fig. 1, video 1), with a peak intensity on unloading all the more intense than the load is maintained long. Preliminary uniaxial compression tests (not reported here) up to 26, 37 and 50 MPa showed that the intensity of the mechano-luminescence peak on unloading almost solely depends on the duration of the plateau at the maximum load, regardless of the value of the maximum stress. Therefore, a stress of 26 MPa, that is small enough to avoid premature failure upon loading, was chosen to investigate this phenomenon. Two striking features of the "unloading" peak are that (1) it is much more intense when the load is applied concomitantly with the UV irradiation (Fig. 2), and (2) it does not show up when the material is loaded in an isostatic manner. In this latter case, a rather intense peak is observed as soon as the pressure is applied. The peak intensity was found to be almost proportional to the pressure (series of experiments not shown here), and a sudden decrease of the EML intensity is noticed when the pressure is relieved. This suggests that the deviatoric part of the applied stress (i.e. the isochoric contribution associated with shear) is solely responsible for the light emission upon unloading, while the EML observed on loading is chiefly activated by the isostatic component of the stress (i.e. the part inducing volume change), with perhaps some shear contribution too. Furthermore, the intensity keeps decreasing during the load plateau. The delayed detrapping process for the trapped e^−^ has thus much in common, from a phenomenological point of view, with the delayed elastic behavior of viscoelastic media (as for creep anelasticity and stress relaxation). The insets in Figs. 1, 3 show the specimen before loading and at the maximum load respectively. When the material is back to zero stress, phosphorescence relays on and the curve gets back to the phosphorescence background (with some delay though), as will be discussed in the next section.

### The hydraulic circuit analogy

**Figure 4:**
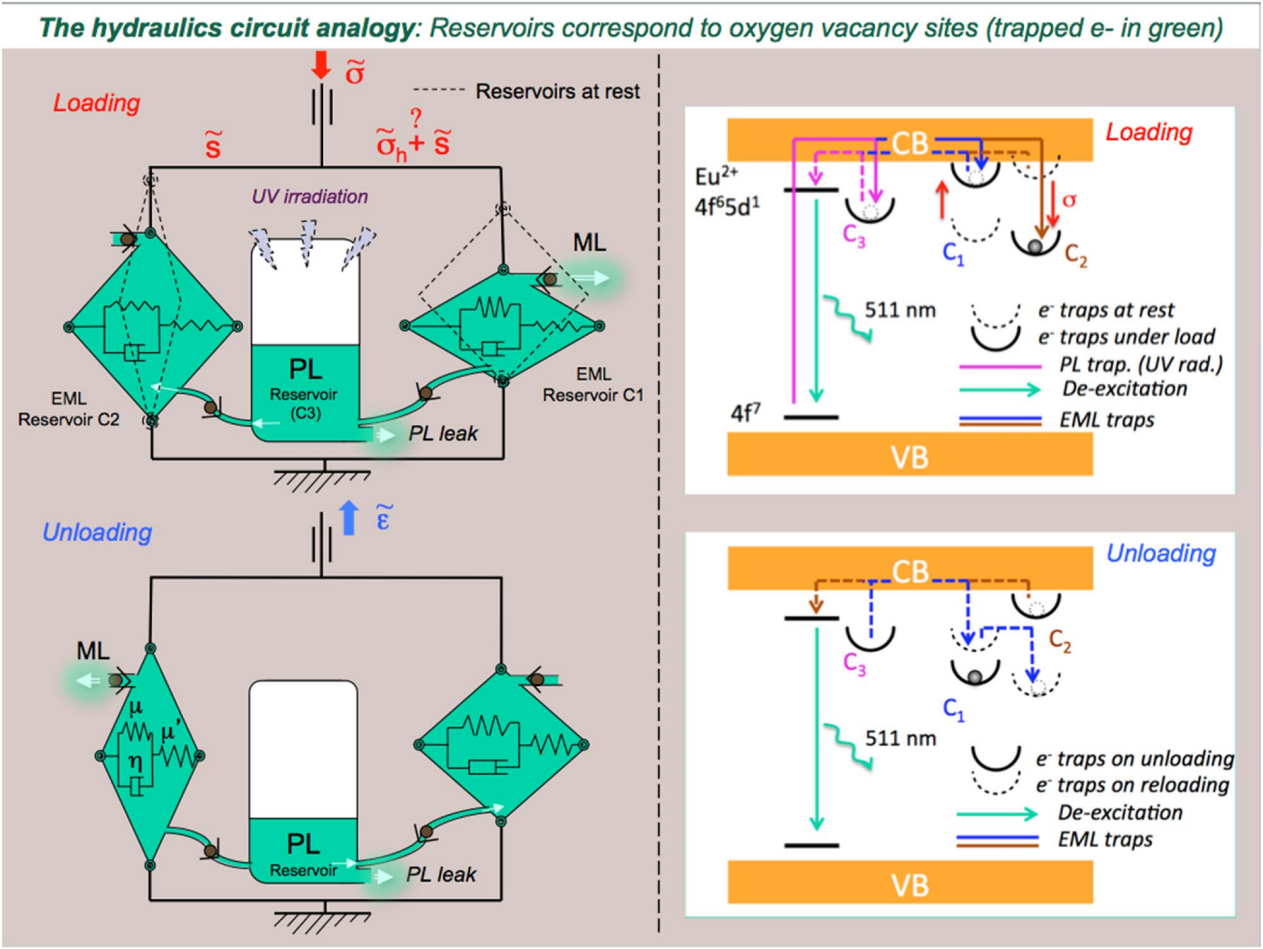
The hydraulic circuit analogy. The e^−^ trapping sites are associated with the potential wells C_1_, C_2_ and C_3_ in the schematic drawings shown on the right. In contrast to C_3_, C_1_ and C_2_ are load-dependent traps that are activated (e^−^ detrapping process) on loading and unloading respectively. But C_2_ is only active if the stress tensor has a non-zero deviatoric part. C_3_ is the e^−^ trapping energy level responsible for the phosphorescence effect, and is load independent. UV light promotes the filling-up of C_3_, i.e. of the central reservoir in the left-hand side drawing. Under loading, the C_1_ tank empties, while the C_2_ one fills up, and vice-versa on unloading. C_1_ and C_2_ tanks are built on articulated frames (black circles are patella joints) kinematically governed by Zener cells.

The obtained composite behaves as a photonic sponge that is able to store some e^−^ in traps deeper than the primary traps responsible for the fluorescence effect, so as to prevent them from reaching the conduction band and relax through photon emission within the experimental time scale (few hours). A mechanical loading may induce a shift of the energy levels of these secondary traps closer to the band gap, hence leading to detrapping and mechanoluminescence. At this stage of our understanding, the hydraulics circuit presented in Fig. 4 provides a faithful description of the complex mechano-optical coupling observed in the studied EML material. The central reservoir (C_3_) corresponds to the overall available trapped e^−^ at oxygen vacancy sites thanks to the prior UV irradiation, and inducing phosphorescence. The leak at the bottom of the reservoir accounts for phosphorescence where, in contrast to Toricelli relation assuming a linear decrease of the volumetric flux with time, phosphorescence results from a thermally activated de-trapping process, and thus follows an exponential decay. Then, in regard with the positioning of the one way valves, the C_1_ tank empties on loading while the C_2_ one fills up, and vice-versa upon unloading. Interestingly, if UV irradiation is performed under load, then the C_2_ tank may be filled from the beginning, provided the stress is not purely hydrostatic. The model is completed by considering that the kinematics of the filling and emptying of the tanks is governed by a Zener-type rheological cell^18^, which allows to take into account the delayed effects that are observed (the tanks are thus built as articulated frames). The Zener model is the simplest one able to account for both stress-imposed (creep) and strain-imposed (relaxation) (or strain-controlled) experiments. Besides, it leads to an exponential form for the decay, in agreement with the experimental observation for PL as well as for EML. A Zener cell (inset in the bottom left tank in Fig. 4) consists of a Kelvin–Voigt cell (a spring of stiffness µ in parallel with a dashpot of viscosity η) in series with a spring (stiffness µ′), and is thus characterized by a time constant τ = η/µ. The schematic drawing of the e^−^ energy levels on the right hand side in Fig. 4 illustrates the trapping-detrapping mechanisms associated with the hydraulic circuit. Under UV irradiation, the valence electrons on the 4f. energy level of Eu^2+^ jump to the conduction band (CB), inducing the Eu^3+^ state, and may be further trapped in defect states. Trapped electrons in defect levels close to CB (C_3_ population) can then (1) relax to the 4f^6^5d^1^ level thanks to thermal activation, with the corresponding emission of photons (PL); or (2) fall in deeper energy levels (C_1_ population at rest or C_2_ one under load), which require the assistance of mechanical loading to activate the de-trapping process within the experimental time-scale. C_2_ approaches CB at rest, so that e^−^ are preferentially filling up C_1_, in agreement with the fact that the C_2_ tank is close at rest in the analogy with hydraulics. Nevertheless, C_2_ becomes deeper under mechanical loading and thus attracts e^−^, whereas C_1_ gets concomitantly closer to CB and relieves its e^−^ through CB, and vice-versa upon unloading, so that light is emitted thanks to C_1_ and C_2_ detrapping on loading and unloading respectively, while C_3_ is almost load-independent. This corresponds to the emptying of the C_1_ tank on loading while the C_2_ one fills up in the hydraulics analogy. The absence of a peak on reloading (at t = 1575 s) when UV irradiation was performed under load (Fig. 2), but an increase of the EML intensity during a subsequent unloading (at t = 1600 s), suggests that C_1_ was empty since the beginning of the experiment (because under load C_1_ is close to CB), and that C_2_ was re-filled during the reloading, likely from C_3_ but possibly with a transit through C_1_ (blue arrow in the bottom right drawing in Fig. 4), which gets deeper on unloading. In the case of hydrostatic loading (Fig. 3), unloading results in the filling of the C_1_ reservoir, while C_2_ is inoperant because of the lack of deviatoric contribution (no shear). Therefore, the intensity of the emitted light decreases upon the relief of the pressure, as the filling of C_1_ predominates over the other processes. Then, when the material is back to zero stress, phosphorescence relays on and the curve tends toward the phosphorescence background (with some delay though). Uniaxial testing (compression or tension) combines both hydrostic and shear components. Hence, in the case of uniaxial compression, the presence of some trapped e^−^ in the C_2_ potential well (and associated reservoir in hydraulic circuit analogy) gives birth to a peak upon unloading, and this is the predominant effect until the load approaches zero and that again the emission resumes to its phosphorescence decay. The delayed response of the Zener model also accounts for the dependence of the unloading peak intensity on the loading duration. The U-shape form at C_1_, C_2_ and C_3_ traps were drawn to recall that in all cases detrapping is a thermally activated process too. Beside the ability of the hydraulic circuit analogy to depict all observed phenomena related to the mechanics to light conversion, such analogy opens the way toward a constitutive EML law, and allows for a quantitative modeling of mechano-optical coupling. The creep compliance (J(t) = ε(t)/σ°, where ε(t) is the time-dependent strain stemming from an applied stress σ° (constant) associated with the Zener cell is written: J(t) = 1/(2µ′)[1 + (µ′/µ)(1−exp(−µt/η))]. This retardation function is used to account for the fact that the EML intensity decreases slowly once the stress is suppressed, in analogy with the delayed response of a viscoelastic material. A hereditary mechanical power (P_m_(t)) is calculated at time t from the whole loading history. The EML intensity is proportional to P_m_(t) and to the fraction of remaining trapped e^−^: ${I}_{EML} \propto {P}_{m}(t)^{\left({N}_{te}^{0}-{N}_{de}(t)\right)/{N}_{te}^{0}}$, where ${N}_{te}^{0}$ is the initial number of trapped e^−^ (following UV irradiation) and ${N}_{de}(t)$ is the number of detrapped e^−^ at time t (α stands for proportional). This latter equation can be readily integrated and the result is expressed as: ${I}_{EML}= C \left\{1-exp\left[-C^{\prime}{\int }_{0}^{t}{P}_{m}(t^{\prime})dt^{\prime}\right]\right\}$, where C and C' are positive constants. The dashed lines in Fig. 1 show the result of the modeling when the parameters of the Zener model are optimized to fit the experimental data using the Microsoft excel solver tool, and assuming that the isostatic part, σ_h_, of the stress tensor $\stackrel{\sim }{\sigma }$ gives no contribution to the light emission peak occurring on unloading, which is hence solely governed by the deviatoric part, $\stackrel{\sim }{s}$, and by the shear characteristics. Values of 2 GPa, 7 × 10^10^ Pa s, 6 × 10^–5^ and 8 × 10^–6^ Pa^−1^ were obtained for µ, η, C, and C′ respectively, by optimization of the curve fitting, taking 20.5 GPa for µ′, which is the actual value for the shear modulus of the NaPOLi glass matrix. These values yield a time constant (τ_EML_ = η/µ) of 35 s for the EML intensity decay, which is close to the time constant for the phosphorescence (τ_PL_ = 37 s), after 30 s UV irradiation in ambient conditions, by fitting the decay by a stretched exponential expression (exp[(−t/τ_PL_)^β^], with β = 0.5^19^. This suggests that the mechanical loading is triggering a de-trapping mechanism by bringing the potential energy level of the relevant traps close to the one associated with the natural phosphorescence.

In uniaxial testing (stress σ), the stored elastic energy per unit volume is σ^2^/(2E), where E is Young's modulus. This energy is the sum of two contributions, namely the energy involved in the volume change, σ^2^(1–2ν)/(6E), and the one associated with the shape change, σ^2^(1 + ν)/(3E), where ν is Poisson's ratio. This makes a fraction of 2(1 + ν)/3 of the energy available for the shear driven unloading effect. In the present case, ν = 0.29, and 86% of the energy is supposed to be responsible for the unloading effect. This number goes down to zero in hydrostatic pressure and up to 100% in simple torsion.

### The DFT analysis

In order to confirm the present picture, we must first identify the chemical nature of the defect levels C_1_, C_2_ and C_3_ and understand their behavior under mechanical loading and unloading. In such a system, based on SrAl_2_O_4_:Eu,Dy particles, it is already well-known that the defects responsible for the e^−^ trapping and detrapping are the oxygen vacancies. However, in its ambient pressure monoclinic phase, SrAl_2_O_4_ (referred to as SAO) exhibits 8 unequal oxygen sites (video 2). It is thus not trivial to identify which oxygen sites are responsible for the three defect levels C_1_, C_2_ and C_3_.

**Figure 5:**
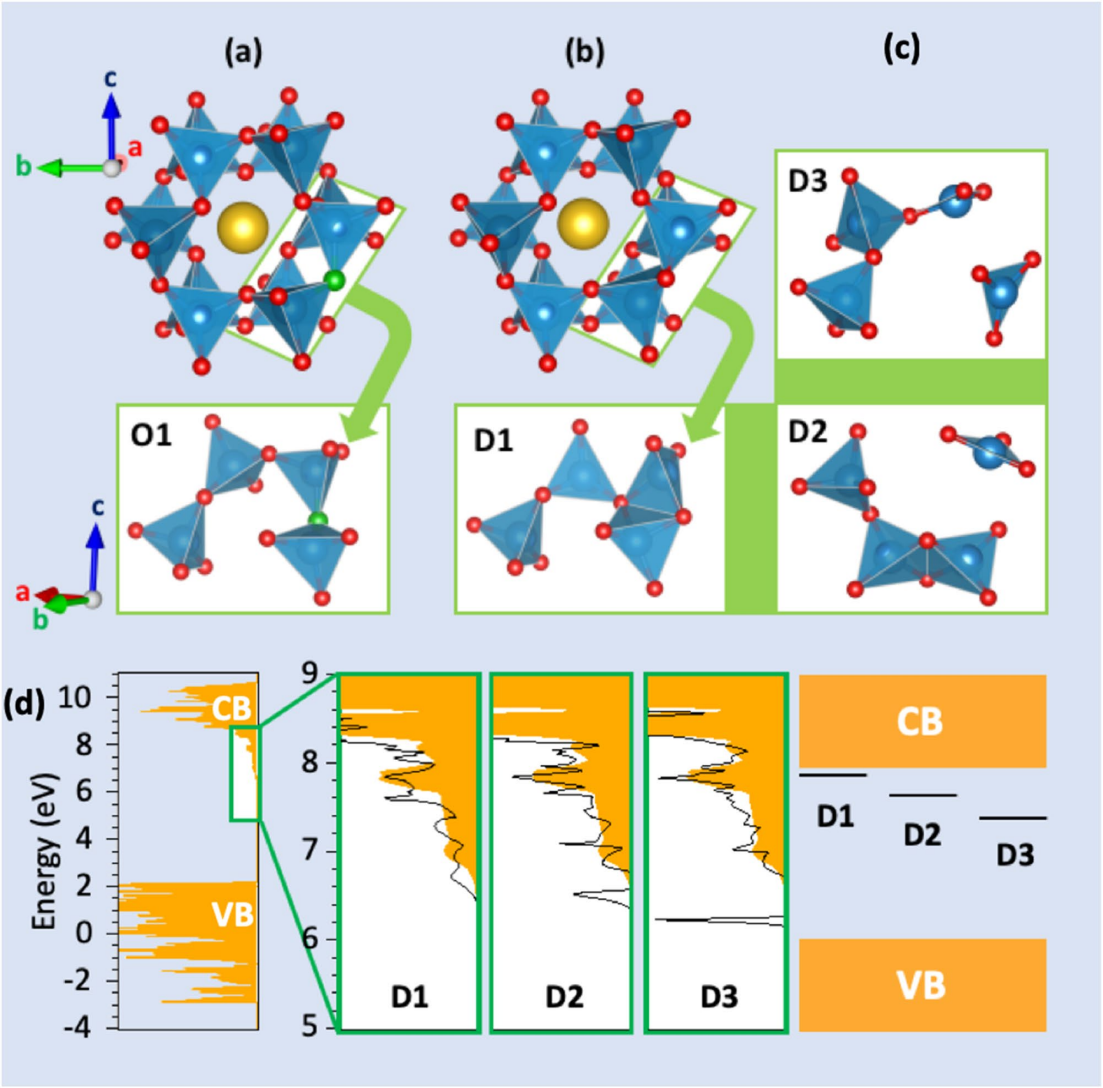
SrAl_2_O_4_ (SAO) crystal structure before and after the formation of an oxygen vacancy (${V}_{O}^{\bullet }$) and its related effects on the electronic structure. The SAO relaxed atomic structure before (**a**) and after (**b**) the formation of V_O_ on the O_1_ site is schematically represented (see also video 2, on line). O, Al and Sr atoms are represented in red, blue and yellow, respectively (O_1_ highlighted in green). (**c**) Three types of local arrangements around V_O_ can be formed and corresponds to D_1_, D_2_ and D_3_ defects. (**d**) The SAO density of states (DOS) is represented in orange and the DOS of D_1_, D_2_ and D_3_ are superposed to evidence the position of the related defect energy level in the band gap. A band diagram is provided to summarize the DFT results obtained for oxygen vacancies containing one electron.

**Figure 6:**
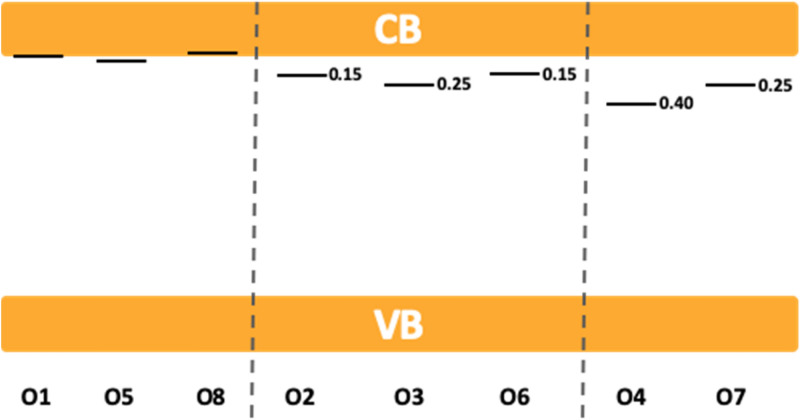
Band diagrams corresponding to the 8 inequivalent oxygen vacancies containing one electron at ambient pressure. The energy position with respect to the bottom of the conduction band is given when the level is inside the band gap. The related SrAl_2_O_4_ monoclinic crystal and the 8 inequivalent oxygen sites are depicted on a video (on line).

The crystallographic structure of SAO consists of a tridimensional network of corner-sharing AlO_4_ tetrahedra, thus forming cages in which Sr atoms are inserted (Fig. 5). This peculiar entanglement makes SAO very sensitive to any structural change because (1) some oxygen sites, despite being separated by 3–4 Al–O bonds, are in fact very close in space, and (2) oxygen vacancies are expected to induce long-range modifications within these AlO_4_ rings. Therefore, 8 models needed to be investigated, each of them containing an oxygen vacancy, V_O_, on a different oxygen site. Before discussing our results, it should be mentioned that Finley et al.^9^ have recently investigated the role of intrinsic defects in SAO using DFT calculations. In particular, their estimation of the formation energies related to the creation of an oxygen vacancy on the 8 unequal sites led them to restrict the thermodynamic analysis to only 4 oxygen sites. However, the energy difference among the 8 oxygen vacancies is at most 0.1 eV, which is very small considering the error bar of the method and the high-temperature conditions required to produce SAO. Hence, we find necessary to evaluate the thermodynamic properties for the 8 sites. An oxygen vacancy containing one electron has been considered to properly describe the potentially trapped e^−^ in the related defect level, due to UV excitation (Fig. 6). It is written ${V}_{O}^{\bullet }$ in the Kröger–Vink notation. A (2a,2b,3c) supercell was used to avoid spurious interactions between the periodic images of ${V}_{O}^{\bullet }$. After relaxation of the atomic positions, the 8 models lead to only 3 different local rearrangements (D_1_, D_2_ and D_3_ in Fig. 5). More specifically, creating a vacancy on O_1_, O_2_ and O_3_ sites led to one atomic rearrangement, named D_1_. Similarly, O_2_, O_3_ and O_6_ led to D_2_, and O_4_ and O_7_ to D_3_. An illustration is provided for the O_1_ site (green color) before (Fig. 5a) and after the creation of the vacancy (Fig. 5b). The structural relaxation in this specific situation induces the formation of an edge-sharing connection between 2 AlO_4_ tetrahedra (Fig. 5c). Then, the relaxed configuration following the structural rearrangements in the vicinity of the vacancy sites give birth either to a tetrahedral environment (D_1_), or to a trigonal planar (TP) environment (D_2_), or to 2 TP environments (D_3_), with edge-sharing connections occurring in the presence of oxygen vacancies in the case of D_1_ and D_2_ only (Fig. 5c). Such structural variations lead to different defect energy levels in the band gap (Fig. 5d). Indeed, for an oxygen vacancy occupied with one electron, our calculations show that D_1_, D_2_ and D_3_ defect levels are expected at about 0.05, 0.20 and 0.35 eV from the conduction band, respectively. We can conclude from the present DFT calculations that the 8 unequal oxygen sites generate only 3 different defect levels upon the creation of oxygen vacancies, which are characterized by 3 different local atomic arrangements and associated potential energy levels. This picture corroborates the analysis of the EML experimental data, and in particular the existence of 3 independent reservoirs in the hydraulics analogy.

Then, the behavior of the material under mechanical loading was addressed and simulated in both hydrostatic pressure and uniaxial loading cases. It should be noted that the simulated mechanical stresses were purposely large, not only to exacerbate the changes in the energy levels associated with the identified defects, but also because large stresses (stress concentration) may also develop at particle sites due to sharp contact loading stemming from particle/particle interactions. The hydrostatic pressure was set by uniformly reducing the lattice volume by 2%. This value corresponds to a pressure of 6 GPa (after a Birch–Murnaghan^20^ fit of the E = f(V) curve). Such a large pressure was not found to affect the crystallographic structure, nor the local environment of all possible oxygen vacancies with respect to their ground state configuration (ambient pressure). As a consequence, no significant change was observed regarding the energy level of the electronic traps. Nevertheless, the application of a shear loading, by imposing a 0.8 Å in plane displacement over a 8 Å interplanar distance (i.e. an angular distortion of 0.1 resulting in a shear stress of ~ 2 GPa) induces a noticeable distortion of the unit cell and thus, of the orbital interactions within the bonds. Here, six shear displacements are possible, i.e. (a,b) plane may glide either along the a or the b direction, and similarly for (b,c) and (a,c) planes. We found that shearing the structure by a displacement of the (b,c) plane along the c direction is the most energetically favorable, as it induces only a variation of the monoclinic angle. This uniaxial distortion is sufficient to induce an electric polarization in the SAO crystal, which is known to be piezoelectric. This polarization is likely to act as a driving force for the detrapping processes^10^.

**Figure 7:**
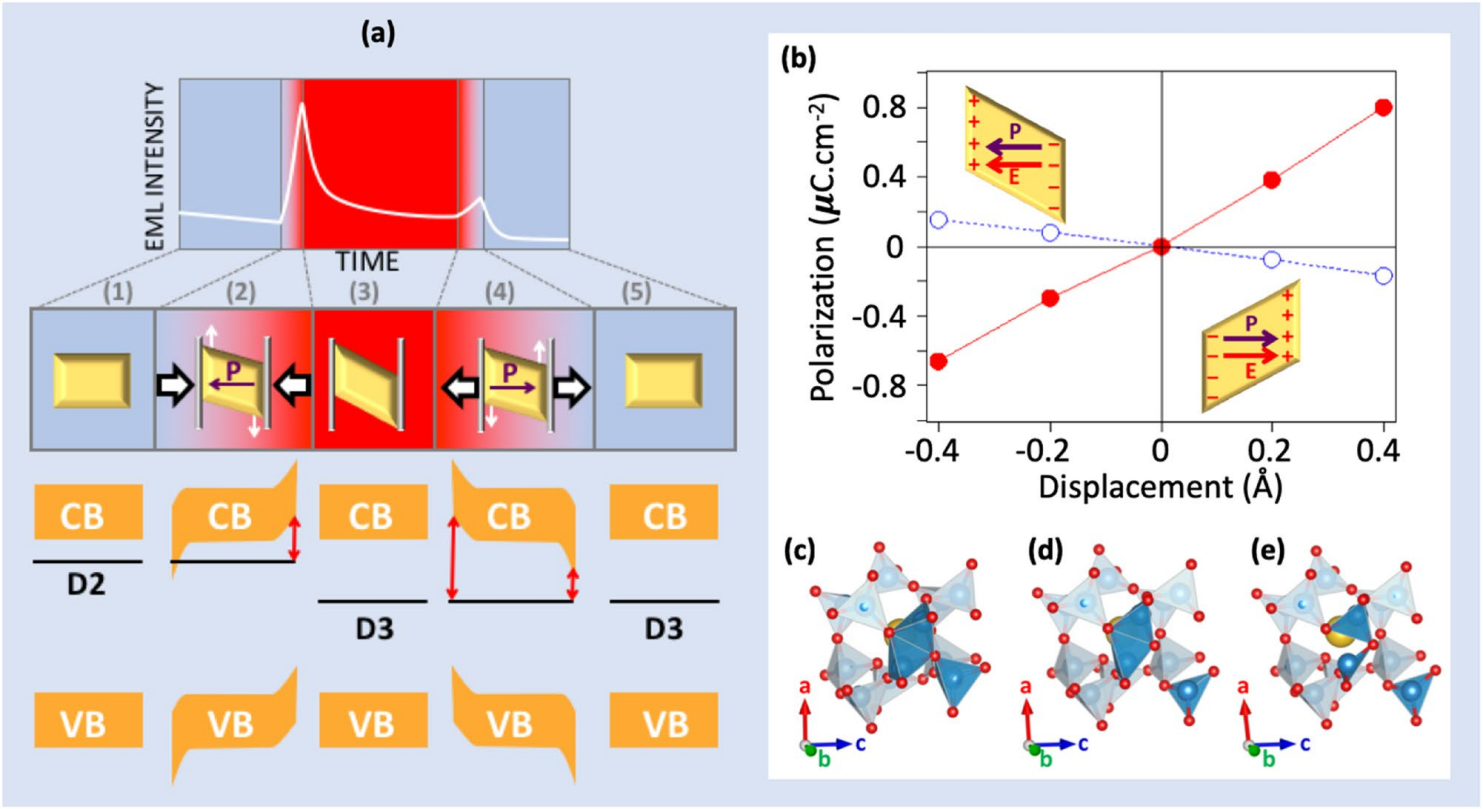
Effect of a mechanical loading on SAO particles. (**a**) The EML intensity changes under uniaxial loading and unloading can be decomposed in 5 steps (detailed in the text). (**b**) The calculated polarization for a displacement of the (**b**,**c**) plane along the c direction is represented without and with structural relaxation, in blue dashed line and red line, respectively. The relaxed atomic structure of SAO for a displacement of − 0.8 (**c**), 0 (**d**) and + 0.8 (**e**) Å of the (**b**,**c**) plane along the c direction is represented. The yellow parallelogram represents one nanoparticle that locally feels a shearing when an uniaxial loading is applied to the composite material.

The schematic drawings shown in Fig. 7a illustrate the five steps associated with the EML phenomenon during a loading-plateau-unloading cycle performed in uniaxial compression on a composite consisting of SAO particles embedded in the NaPOLi glass. As was already recalled at the end of the former section, a uniaxial loading involves the combination of hydrostatic and deviatoric components, and this latter contribution is the source for the distortion (by shear) of the SAO crystals (such as plastic flow occurs along sliding planes shifted to the load axis in ductile metals in compression). The five steps are as follows: (1) the SAO is under ambient pressure, in its ground state atomic structure and is exposed to UV radiations; (2) a uniaxial pressure is progressively applied leading to a deformation of the particle, including an angular distorsion that induces a polarization further leading to a bending of the conduction and valence bands; (3) the SAO is under a constant pressure and remains in a metastable distorted atomic structure. No polarization and thus no band bending shows up during this stage, but local atomic rearrangements could lead to a conversion of the defects, from D_2_ to D_3_ (deeper) in the present case; (4) the pressure is removed and the SAO progressively returns to its ground states cell parameters. It leads to a piezoelectric polarization and to band bending, which are both reversed compared to the situation in step (2). As a matter of fact, the D_3_ population gets closer to the CB and detrapping occurs; (5) the SAO is back to ambient conditions and depending on the history of the atomic restructuration during all the process, and remaining trapped e^−^ are unlikely to provide any luminescence (it was observed that a re-loading performed weeks after the first experiment, the material being kept in complete darkness in the time interval, would still activate some residual EML phenomenon).

The evolution of the electric polarization calculated for SAO under a displacement of the (b,c) plane from − 0.4 to + 0.4 Å along the c direction is shown in Fig. 7b. These estimations were done using the Berry Phase formalism^20,21^. A displacement of + 0.4 Å along the c direction of the (b,c) plane (i.e. and increasing the monoclinic angle from 93.5° to 96.3°) is found to induce a spontaneous polarization of + 0.80 µC cm^−2^. Such a polarization arises during the deformation of the SAO and leads to the appearance of an electric field, which bends the e^−^ bands. As a consequence, electronic traps may get closer to the conduction band, increasing the detrapping of electrons in the related defect levels. A remarkable finding of the present study is that polarization only arises during the deformation, i.e. when the pressure is changing. Such an out-of-equilibrium condition appears in steps (2) and (4), during loading and unloading, respectively. In contrast, no polarization, and thus no band bending, is observed in step (3), which corresponds to the SAO crystal with a fixed deformation under constant load. Our calculations evidenced that at this stage it is possible to observe a defect conversion and thus a displacement of the related energy level in the band gap. For instance, applying a displacement of the (b,c) plane of − 0.8 Å and + 0.8 Å along c for the supercell containing a D_2_ defect (Fig. 7d), respectively leads to a conversion to D_1_ (Fig. 7c) and D_3_ (Fig. 7e) defects.

## Conclusion and perspectives

The EML phenomenon in a glass matrix particulate composite containing SAO particles was addressed both experimentally and theoretically. From the point of view of mechanics, the deviatoric part of the stress field is found responsible for the EML intensity observed upon unloading and the behavior could be modeled by means of an analogy with hydraulic circuits involving three reservoirs representing the trapped e^−^ population. DFT calculations provided links between these reservoirs and the three types of defects identified at oxygen vacancy sites. This structural analysis showed that the shear distortion of the particles gives birth to the light emission and provides an explanation for the regain of intensity observed during the transient loading and unloading regimes. This latter contribution would stem from the bending of the CB thanks to the polarization of the crystal, which moves the trapped e^−^ closer to the CB. This paves the way to a physically sound constitutive law for EML phenomenon, and to the design of more EML efficient materials.

## Supplementary information

Supplementary Information 1.

Supplementary Video 1.

Supplementary Video 2.
