# Supplementary material for: Mechanics and physics of a glass/particles photonic sponge
Source: Sci Rep. 2020 Nov 11;10:19495. doi: 10.1038/s41598-020-75504-9 (PMC7658237; doi:10.1038/s41598-020-75504-9)
Supplement: Supplementary file 1 — Supplementary Information 1. [file 41598_2020_75504_MOESM1_ESM.docx]

**Mechanics and Physics of a Glass/Particles Photonic Sponge**

***M. Dubernet^1^, E. Bruyer^2^, Y. Gueguen^1^, P. Houizot^1^, J.C. Hameline^1^, X. Rocquefelte^2^ and T. Rouxel^1,*^***

*^1^Physics Institute, IPR, UMR UR1-CNRS 6251, Université de Rennes 1, 35042 Rennes cedex, France*

*^2^Chemistry Institute, ISCR, UMR UR1-CNRS 6226, Université de Rennes 1, 35042 Rennes cedex, France*

**Corresponding author :* [*tanguy.rouxel@univ-rennes1.fr*](mailto:tanguy.rouxel@univ-rennes1.fr)

**Supplementary materials (2 videos)**

**Video 1:** The elasto-mechanoluminescence phenomenon in a glass (matrix) SrAl_2_O_4_:Eu,Dy (particles) composite, in compression.

**Video 2:** The atomic structure of the monoclinic phase of SrAl_2_O_4_ with the eight unequal oxygen vacancy sites.
